# Note from the editors: Celebrating collaboration on World Field Epidemiology Day

**DOI:** 10.2807/1560-7917.ES.2024.29.36.240905m

**Published:** 2024-09-05

**Authors:** Eurosurveillance editorial team

**Affiliations:** 1 European Centre for Disease Prevention and Control (ECDC), Stockholm, Sweden

World Field Epidemiology Day (WFED) is commemorated yearly on 7 September, to raise awareness of the contributions of field epidemiologists around the globe to protect health and safeguard global health security.

This year’s theme is **Collaboration is Essential to Field Epidemiology,** highlighting the importance of collaboration in every realm of field epidemiology, ranging from partnerships with institutes and international organisations, to the cooperation on the ground with people in communities. WFED was launched in 2021 by the Training Programs in Epidemiology and Public Health Interventions Network (TEPHINET).

*Eurosurveillance* has published multiple articles on field epidemiology and drawing from field epidemiology techniques and has commemorated WFED since its inception in 2021 [1]. In this issue, a study by Cunningham et al. on a Shiga toxin-producing *Escherichia coli* infection outbreak in the United Kingdom applied multiple cross-sectoral methods and datasets to identify the source of the outbreak: analytical epidemiological methods, food chain analyses and weather and animal density data. This information was essential to identify contaminated lettuce as the likely source of the outbreak [2].

The European Centre for Disease Prevention and Control (ECDC) runs the European Programme for Intervention Epidemiology Training (EPIET) and the European Programme on Health Microbiology (EUPHEM), which provide work experience and training for epidemiologists in the field in EU/EEA countries.

EPIET and EUPHEM were highlighted in an *Eurosurveillance* article on WFED 2023 [3]. In the same issue, an article by Hahné et al. presented reflections on the evolution of field epidemiology and its future after its unprecedented visibility during the COVID-19 pandemic, based on an analysis of discussions with EPIET and EUPHEM fellows and alumni during a workshop [4].

ECDC also manages the Mediterranean and Black Sea Programme for Intervention Epidemiology Training (MediPIET) programme, which provides opportunities in partner countries in the Mediterranean and Black Sea and was the focus of the editorial for last year’s WFED [5].
